# A translocator protein 18 kDa agonist protects against cerebral ischemia/reperfusion injury

**DOI:** 10.1186/s12974-017-0921-7

**Published:** 2017-07-28

**Authors:** Han-Dong Li, Minshu Li, Elaine Shi, Wei-Na Jin, Kristofer Wood, Rayna Gonzales, Qiang Liu

**Affiliations:** 1 Department of Neurology, Tianjin Neurological Institute, Tianjin Medical University General Hospital, Tianjin, 300052 China; 2 Department of Neurology, Barrow Neurological Institute, St. Joseph’s Hospital and Medical Center, Phoenix, AZ 85013 USA; 3 Department of Basic Medical Sciences, University of Arizona College of Medicine, Phoenix, AZ 85004 USA

**Keywords:** TSPO, Etifoxine, Neuroinflammation, Cerebral ischemia

## Abstract

**Background:**

Cerebral ischemia is a leading cause of death and disability with limited treatment options. Although inflammatory and immune responses participate in ischemic brain injury, the molecular regulators of neuroinflammation after ischemia remain to be defined. Translocator protein 18 kDa (TSPO) mainly localized to the mitochondrial outer membrane is predominantly expressed in glia within the central nervous system during inflammatory conditions. This study investigated the effect of a TSPO agonist, etifoxine, on neuroinflammation and brain injury after ischemia/reperfusion.

**Methods:**

We used a mouse model of middle cerebral artery occlusion (MCAO) to examine the therapeutic potential and mechanisms of neuroprotection by etifoxine.

**Results:**

TSPO was upregulated in Iba1^+^ or CD11b^+^CD45^int^ cells from mice subjected to MCAO and reperfusion. Etifoxine significantly attenuated neurodeficits and infarct volume after MCAO and reperfusion. The attenuation was pronounced in mice subjected to 30, 60, or 90 min MCAO. Etifoxine reduced production of pro-inflammatory factors in the ischemic brain. In addition, etifoxine treatment led to decreased expression of interleukin-1β, interleukin-6, tumor necrosis factor-α, and inducible nitric oxide synthase by microglia. Notably, the benefit of etifoxine against brain infarction was ablated in mice depleted of microglia using a colony-stimulating factor 1 receptor inhibitor.

**Conclusions:**

These findings indicate that the TSPO agonist, etifoxine, reduces neuroinflammation and brain injury after ischemia/reperfusion. The therapeutic potential of targeting TSPO requires further investigations in ischemic stroke.

## Background

Cerebral ischemia is a leading cause of death and disability worldwide. Despite the advances of reperfusion therapy, only very limited number of patients with ischemic stroke can benefit from these treatments. Owing to the failure of many clinical neuroprotective trials, there is an urgent yet unmet need for a better understanding of the pathogenic processes triggered by brain ischemia and reperfusion. Inflammatory and immune responses are key elements involved in the onset and progression of stroke [1–3]. Therefore, targeting the regulatory mechanisms of brain inflammation has high therapeutic potential and is being actively explored.

The translocator protein 18 kDa (TSPO) is a trans-membrane protein that is primarily localized on the outer mitochondrial membrane [4]. TSPO was first identified as a peripheral-type benzodiazepine receptor and represents a multiprotein complex implicated in the regulation of steroid genesis, inflammation, and cell proliferation [5, 6]. In the brain, TSPO is predominantly expressed in glial cells [7]. Accumulating evidence has demonstrated increased TSPO expression during several neuroinflammatory conditions such as intracerebral hemorrhage and multiple sclerosis [8–11], suggesting that targeting TSPO may be a viable approach to limiting neuroinflammation and brain injury [12]. However, the cell types that express TSPO after brain ischemia and their involvement in brain injury remain unclear.

Etifoxine is a clinically available, high-affinity TSPO agonist [4]. Previous studies have shown beneficial effects of etifoxine and other TSPO ligands to modulate the microglial response and promote neural survival after several types of central nervous system (CNS) insults [10, 13–15]. To further determine the potential neuroprotective and anti-inflammatory effects of etifoxine treatment on ischemic brain injury, we examined the efficacy and mechanisms of action by etifoxine in mice subjected to middle cerebral artery occlusion (MCAO) and reperfusion.

## Methods

### Animals

All animal experiments were approved by the Institutional Animal Care and Use Committees of Barrow Neurologic Institute and Tianjin Medical University General Hospital. This study was conducted in accordance with the National Institutes of Health (NIH; Bethesda, MD, USA) Guide for the Care and Use of Laboratory Animals, and experiments were designed, performed, and reported according to the Animal Research: Reporting of *In Vivo* Experiments guidelines. Male C57BL/6 mice (7–8 weeks old, 20–25 g body weight) were purchased from the Charles River Laboratories (Wilmington, MA, USA). Animals were housed in animal facilities under standardized light–dark cycle, and provided with free access to food and water. Animals were randomly divided and assigned to experimental groups.

### Middle cerebral artery occlusion (MCAO) model

Transient MCAO model was induced by 60 min focal cerebral ischemia and reperfusion using a filament method, as we previously described [16–18]. Briefly, mice were anesthetized by inhalation of 3.5% isoflurane and kept by inhalation of 1.0–2.0% isoflurane in 70% N_2_O and 30% O^2^ using a face mask. A 6–0 nylon filament with rounded tip was insert into the right MCA to occlusion for 60 min. Reperfusion was established when the filament was withdrawn back to the common carotid artery. Laser Doppler probe (model P10, Moor Instruments, Wilmington, DE) was used to monitor the cerebral blood flow (CBF) for 5 min both before and after MCAO, as well as during reperfusion for 5 min. Relative CBF post reperfusion had to rise to at least 50% of preischemic levels in order for mice to be included for further analyses. During surgery procedures, body temperature was maintained by an electric warming blanket. The sham-operated group mice underwent identical surgical procedures except that the filament was not advanced to the MCA. 2,3,5-Triphenyltetrazolium chloride (TTC) staining was used to measure the infarct volume at day 1 and day 3 after MCAO and reperfusion.

### Drug administration

Etifoxine was dissolved in 1% Tween 80 in 0.9% NaCl solution, as previously described [10, 19]. Mice were given etifoxine (Sigma, St. Louis, MO, USA) at the dose of 50 mg/kg by i.p. injection at indicated time points after MCAO and reperfusion. Mice that received an equal volume of vehicle (0.9% NaCl solution containing 1% Tween 80) were used as vehicle controls. For the microglia depletion experiment, PLX3397 (Selleckchem, Houston, TX) was given by oral gavage at 40 mg/kg/day to each mouse for 21 days prior to MCAO. Drug treatment was continued until the end of experiments as previously described [20].

### 2,3,5-Triphenyltetrazolium chloride (TTC) staining

At days 1 and 3 after MCAO and reperfusion, TTC staining was performed to evaluate infarct volume. Mouse whole brains were obtained rapidly after PBS perfusion. Following exposure for 5 min at −20 °C, frozen whole brains were cut into 1-mm thick coronal slices starting at 1 mm from the frontal tips. Two percent (*v*/*v*) TTC solution (Sigma, St. Louis, MO, USA) was used to stain brain sections for 20 min at 37 °C. Infarct areas were determined as absence of TTC stains and were quantified by Image Pro Plus analysis as previously described [16, 21].

### Neurological function assessment

At days 1 or 3 after MCAO and during reperfusion, the modified Neurological Severity Score (mNSS) was adopted to evaluate neurodeficits [22, 23], which comprises a set of terms to assess motor function (muscle and abnormal movement), sensory function (visual, tactile, and proprioceptive), and reflexes (pinna, corneal, and startle reflexes). The range of scores for mNSS is from 0 to 18. The rating scale defined as: a score of 13–18 indicates severe injury, 7–12 indicates moderate injury, and 1–6 indicates mild injury. Mice received a point if they failed to perform a task. Neurological deficit assessment was performed by investigators blinded to the control and MCAO groups as described previously [16–18].

### Flow cytometry

Single cell suspensions were prepared from brain tissues and stained with fluorochrome conjugated antibodies as previously described [16, 18, 24]. All antibodies were purchased from Biolegend accompany (San Diego, CA, USA), unless otherwise indicated. The following antibodies used were CD45 (Cat#103107,30-F11),CD11b (Cat#101228,M1/70), interleukin 6 (IL-6) (Cat#504504, MP5-20 F3), tumor necrosis factor alpha (TNF-α) (Cat#506315, MP6-XT22), and PBR antibody (Ab109497, Abcam, Cambridge, MA, USA). Alexa Fluor®488-labeled donkey anti-rabbit IgG secondary antibody (Invitrogen, Carlsbad, CA, USA) were used for final detection. Flow cytometry was performed on a FACSAria flow cytometer. Data were analyzed using Flow Jo 7.6.1 software.

### Immunostaining

Mice were anesthetized and perfused with 50 ml cold PBS, followed by 30 ml 4% paraformaldehyde perfusion solution. Whole brains were harvested and fixed in 4% paraformaldehyde overnight, then dehydrated with 15 and 30% sucrose. Frozen brains were sectioned (10 μm) and permeabilized with 0.3% Triton X-100 for 20 min. After blocking in 5% goat or donkey serum/PBS solution for 1 h at room temperature, the slices were then incubated with primary antibodies, goat anti-Iba1 (1:500, Abcam, Cambridge, MA, USA), and rabbit anti-PBR (1:200, Abcam, Cambridge, MA, USA) at 4 °C overnight. Following washes x3s in PBS, brain slices were incubated in secondary antibodies: donkey anti-rabbit 594 (1:1000, Invitrogen, Carlsbad, CA, USA) and donkey anti-goat 488 (1:1000, Invitrogen, Carlsbad, CA, USA) for 1 h at room temperature. Finally, the slides were stained with DAPI (Abcam, Cambridge, MA, USA) to counterstain cell nuclei. Images were captured with a microscope (Nikon, C-HGFI, Japan) and data analyzed with Image J (National Institutes of Health, Washington, DC, USA).

### Real-time PCR

At day 1 post MCAO, total mRNA was extracted from the ischemic hemisphere brain tissue using Trizol reagent (Invitrogen, Carlsbad, CA, USA) according to the manual instructions. After determination of the concentration of mRNA, mRNA (1 μg) was reversed transcribed into cDNA using PrimeScript^TM^ RT reagent Kit (TaKaRa, Shiga, Japan). Amplification of gene sequence using SYBR gene polymerase chain reaction (PCR) Master Mix (Roche, Indianapolis, IN, USA) was performed on the Opticon 2 Real-Time PCR Detection System (Bio-Rad). The primers used in our study are listed as follows: TNF-α forward: TATGGCTCAGGGTCCAACTC, TNF-α reverse: GGAAAGCCCATTTGAGTCCT; IL-6 forward: GCTGGTGACAACCACGGCCT, IL-6 reverse: AGCCTCCGACTTGTGAAGTGGT; IL-1β forward: TGCCACCTTTTGACAGTGATG, IL-1β reverse: TGATGTGCTGCTGCGAGATT; iNOS forward: TCCTGGACACGACCCCT, iNOS reverse: CTCTGACTGACACAAGG; IL-10 forward: AAATAAGAGCAAGGCAGTGG, IL-10 reverse: GTCCAGCAGACTAAATACACAC;β-actin forward: CACCCGCGAGTACAACCTTC, β-actin reverse: CCCATACCCACCATCACACC; β-actin served as a reference gene.

### Statistical analysis

Sample size was determined by power analysis using a significance level of *α* = 0.05 with 80% power to detect statistical differences. Power analysis and sample size calculations were determined using SAS 9.1 software (SAS Institute Inc. Cary, NC). The experimental design was based on previous published studies with similar experimental designs [16, 24]. Data were analyzed by investigators blinded to experimental treatments. Data are shown as mean ± SEM. Statistical analyses was performed using Graphpad 6.0 software. Two-tailed unpaired Student’s *t* test was used to determine significance of differences between two groups. One-way ANOVA followed by the Tukey post hoc test was used for comparison of multi-group data. *P* < 0.05 is considered significant.

## Results

### TSPO expression is increased in mice brain following MCAO and reperfusion

**Fig. 1:**
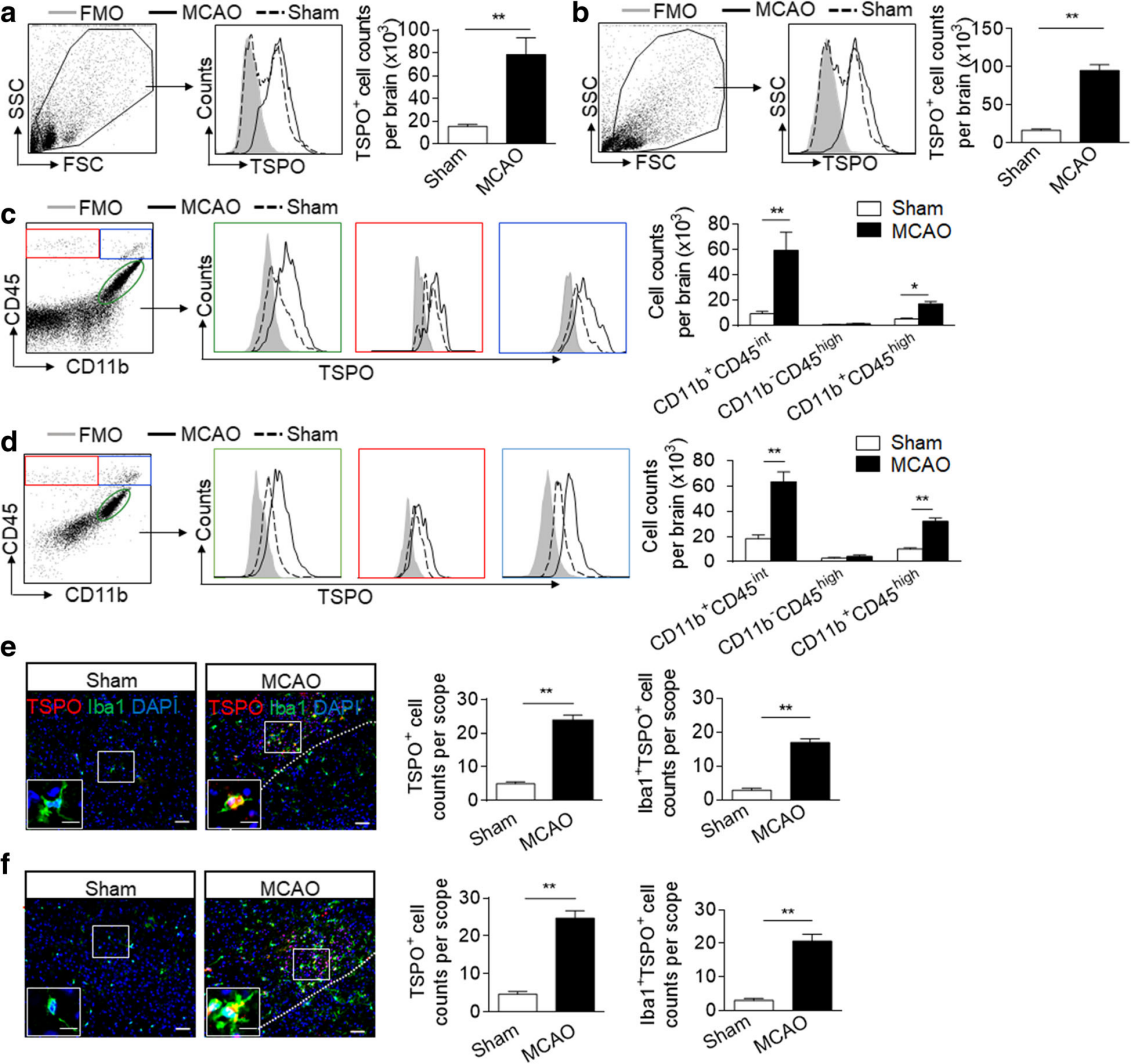
Upregulation of translocator protein 18 kDa (TSPO) expression in the brains of MCAO mice. C57BL/6 mice were subjected to sham operation or 60 min MCAO and reperfusion. Cell suspensions were prepared from whole brain tissues at 24 and 48 h after sham operation or MCAO and reperfusion. **a**, **b** Gating strategy for flow cytometry analysis and bar graph show counts of total TSPO^+^ cells in the brain at 24 (**a**) and 48 h (**b**) after sham operation or MCAO and reperfusion. *n* = 6 per group. **c**, **d** Gating strategy for flow cytometry analysis and bar graph show counts of CD11b^+^CD45^int^TSPO^+^, CD11b^+^CD45^high^TSPO^+^, and CD11b^−^CD45^high^TSPO^+^ cells in the brain at 24 (**c**) and 48 h (**d**) after sham operation or MCAO and reperfusion. *n* = 6 per group. **e**, **f** Immunostaining images and bar graphs show TSPO^+^ and/or Iba1^+^cells in brain slices of mice at 24 (**e**) and 48 h (**f**) after sham operation or MCAO and reperfusion. *Scale bar*: 25 μm, 5 μm (inset). *n* = 18 sections from six mice in each group. Data were presented as mean ± SEM. *P < 0.05, **P < 0.01

To determine the expression profile of TSPO after brain ischemia, we used flow cytometry to measure TSPO-expressing cells from single-cell suspensions isolated from whole brain tissues at 24 and 48 h after MCAO and reperfusion. As shown in Fig. 1a, b, counts of TSPO-expressing cells were significantly increased in cells isolated from MCAO mice versus sham controls. Analysis of gated individual TSPO-expressing cell subsets indicates that microglia (CD11b^+^CD45^int^ cells), to a greater extent than infiltrating leukocytes (CD11b^−^CD45^high^ or CD11b^+^CD45^high^ cells), are the most prominent cell subset expressing TSPO after ischemia (Fig. 1c, d). In addition, we stained the brain sections from mice subjected to MCAO or sham procedures (Fig. 1d, e). A significant increase of TSPO-expressing cells was seen within peri-infarct areas as compared to sham controls (Fig. 1d, e). The expression of TSPO was mostly seen in Iba1^+^ cells (Fig. 1d, e). These findings show an upregulation of TSPO after brain ischemia and the major cell type expressing TSPO is microglia.

### Etifoxine treatment reduces neurodeficits and infarct volume after brain ischemia

**Fig. 2:**
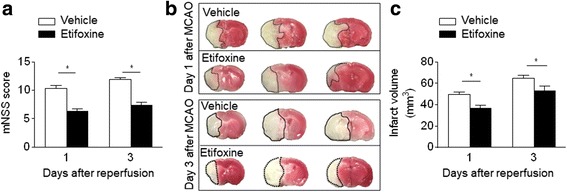
Etifoxine attenuates neurodeficits and brain infarction after MCAO and reperfusion. C57BL/6 mice were given etifoxine (50 mg/kg) or vehicle by daily i.p. injection, starting from immediately after MCAO and reperfusion. At day 1 or day 3 after MCAO and during reperfusion, neurodeficits and infarct volume were measured by modified Neurological Severity Score (mNSS) and TTC staining, respectively. **a** Bar graph shows the modified mNSS in MCAO mice receiving etifoxine or vehicle at indicated time points after reperfusion. **b** TTC-stained brain slices infarct volume from mice receiving etifoxine or vehicle control at indicated time points after MCAO and during reperfusion. **c** Quantification of infarct volume in MCAO mice receiving etifoxine or vehicle control at indicated time points after MCAO and reperfusion. Data were presented as mean ± SEM. *P < 0.05, *n* = 8 per group

To assess whether the TSPO ligand, etifoxine, impacts ischemic brain injury, we examined neurodeficits, and infarct volume in MCAO mice receiving etifoxine or a vehicle control (1% Tween-80, NaCl). Drug treatment was initiated immediately after MCAO and reperfusion. Neurodeficits and infarct volume were assessed at the indicated time points after MCAO and reperfusion. At day 1 or day 3 after MCAO, etifoxine administration significantly reduced neurodeficits (Fig. 2a). This improvement in neurological outcome was concomitant with a reduction in infarct volume in mice receiving etifoxine treatment as compared to vehicle control (Fig. 2b, c).

### Beneficial effects of etifoxine are time dependent

**Fig. 3:**
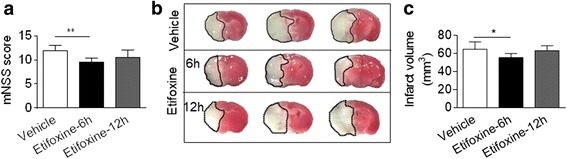
The protective effect of etifoxine is limited within 12 h after MCAO and reperfusion. C57BL/6 mice were given etifoxine (50 mg/kg) or vehicle by daily i.p. injection, starting from 6 or 12 h after MCAO and during reperfusion until the end of experiments. At 72 h after MCAO and reperfusion, neurodeficits and infarct volume were assessed. **a** Quantification of Neurological Severity Score (mNSS) of mice receiving etifoxine or vehicle control at the indicated time after MCAO and reperfusion. **b** TTC-stained brain slices from mice receiving etifoxine or vehicle at 6 and 12 h post MCAO and during reperfusion. **c** Quantification of infarct volume in mice receiving etifoxine or vehicle control at indicated time points after MCAO and reperfusion. Data were presented as mean ± SEM. *P < 0.05, **P < 0.01, *n* = 8 per group

To establish the time window during which etifoxine provides protection against ischemia/reperfusion injury, etifoxine treatment was delayed until 6 or 12 h post MCAO. We found that etifoxine given at 6 h after MCAO attenuated neurodeficits (Fig. 3a) and infarct volume (Fig. 3b, c), similar to that observed when etifoxine was administered immediately after MCAO. However, improvements in neurodeficits by etifoxine was not observed when the drug was administered 12 h post MCAO. These results suggest that the protection of etifoxine treatment was limited within the first 12 h post MCAO and during reperfusion.

### Etifoxine reduces infarct volume in mice subjected to 30 or 90 min MCAO

**Fig. 4:**
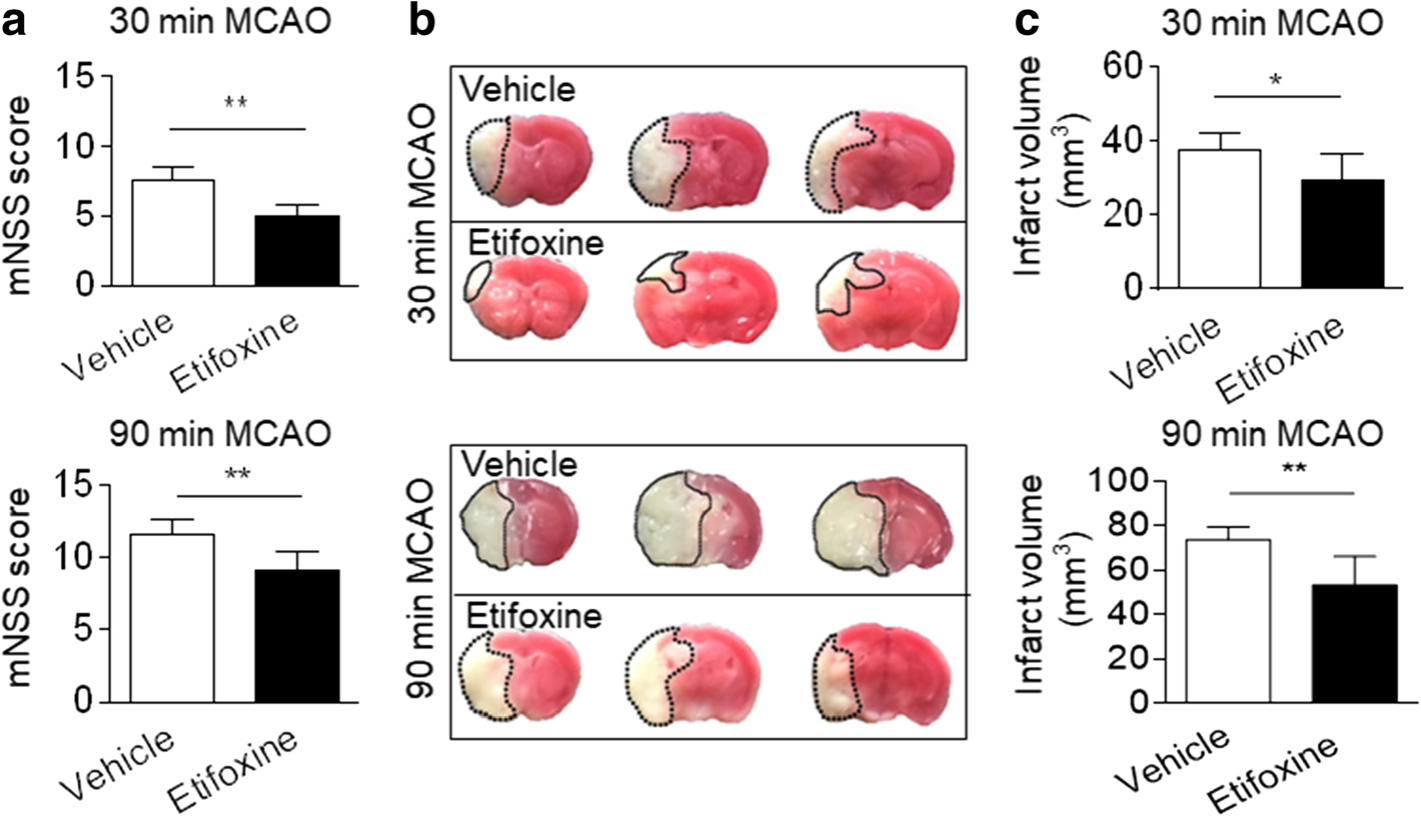
Etifoxine attenuates ischemic brain injury in mice subjected to 30 or 90 min MCAO. C57BL/6 mice were subjected to 30 or 90 min MCAO and reperfusion. Immediately after reperfusion, mice received single i.p. injection of etifoxine (50 mg/kg) or vehicle. At 24 h after MCAO and reperfusion, neurodeficits and infarct volume were measured. **a** Quantification of mNSS in mice receiving etifoxine or vehicle control at 24 h after 30 or 90 min MCAO and reperfusion. **b**. TTC images show infarct in mice receiving etifoxine or vehicle control after 30 or 90 min MCAO and reperfusion. **c** Quantification of infarct volume in MCAO mice with the indicated treatments. Data were presented as mean ± SEM. *P < 0.05, **P < 0.01, *n* = 8 per group

Because infarct volume is a major determinant of brain inflammation and stroke outcome [25], animals that present with various infarct volume may have variability in responses to immune modulation. To assess the responsiveness to etifoxine treatment in animals with different infarct volume, we determined the efficacy of etifoxine in mice subjected to 30 or 90 min MCAO and followed by reperfusion. We observed that etifoxine reduced neurodeficits and infarct volume in mice subjected to either 30 or 90 min MCAO (Fig. 4).

### Etifoxine reduces the production of pro-inflammatory factors after brain ischemia

**Fig. 5:**
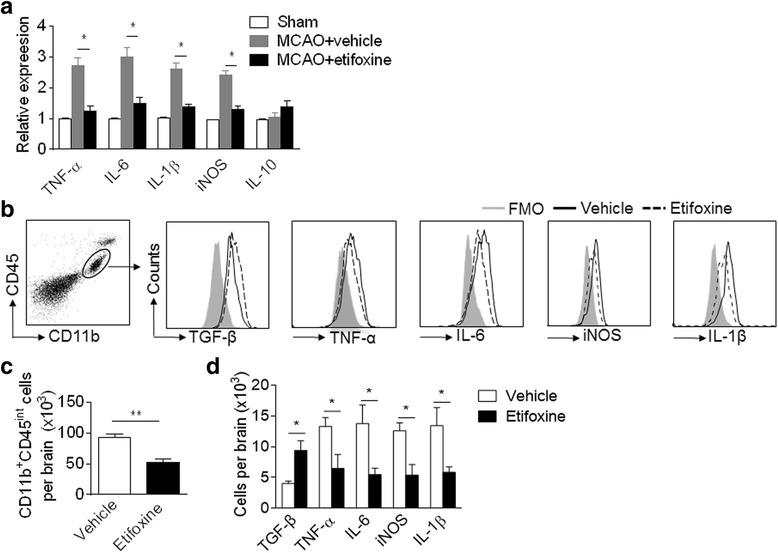
Etifoxine reduces brain inflammation and microglia production of pro-inflammatory factors after MCAO and reperfusion. C57BL/6 mice were subjected to sham operation or 60 min MCAO and reperfusion. Immediately on establishment of reperfusion, mice received a single i.p. injection of etifoxine (50 mg/kg) or vehicle. At 24 h after sham operation or MCAO and reperfusion, whole mouse brain was removed and tissue prepared for cellular and molecular analysis. **a** RT-PCR illustrates cytokine levels in homogenates of the ipsilateral hemisphere brain tissues from indicated groups of mice. Data were presented as mean ± SEM, *P < 0.05, versus vehicle group. *n* = 6 per group. **b** Flow cytometry was performed to measure the number of microglia and their cytokine expression. Flow cytometry plots show gating strategy of microglia and their expression of IL-1β, IL-6, TNF-α, and iNOS. **c** Bar graph shows numbers of total microglia per brain in MCAO mice receiving indicated treatments. **d** Bar graph shows numbers of microglia per brain expressing of IL-1β, IL-6, TNF-α, and iNOS in MCAO mice receiving indicated treatments. Data were presented as mean ± SEM, *P < 0.05, **P < 0.01, *n* = 8 per group

To determine the influence of etifoxine on brain inflammation, we measured the expression of prominent pro-inflammatory cytokines in brain homogenates of MCAO mice receiving etifoxine or vehicle control. At 24 h post MCAO and during reperfusion, we found that etifoxine reduced the production of IL-6 and TNF-α (Fig. 5a). Flow cytometry analysis shows a reduction in microglial cell number after etifoxine treatment, together with the observed decrease in expression of TNF-α and IL-6 (Fig. 5b–d). Thus, these results suggest that etifoxine reduces brain inflammation and microglial production of pro-inflammatory factors after ischemia.

### The protective effect of etifoxine against ischemic brain injury involves microglia

**Fig. 6:**
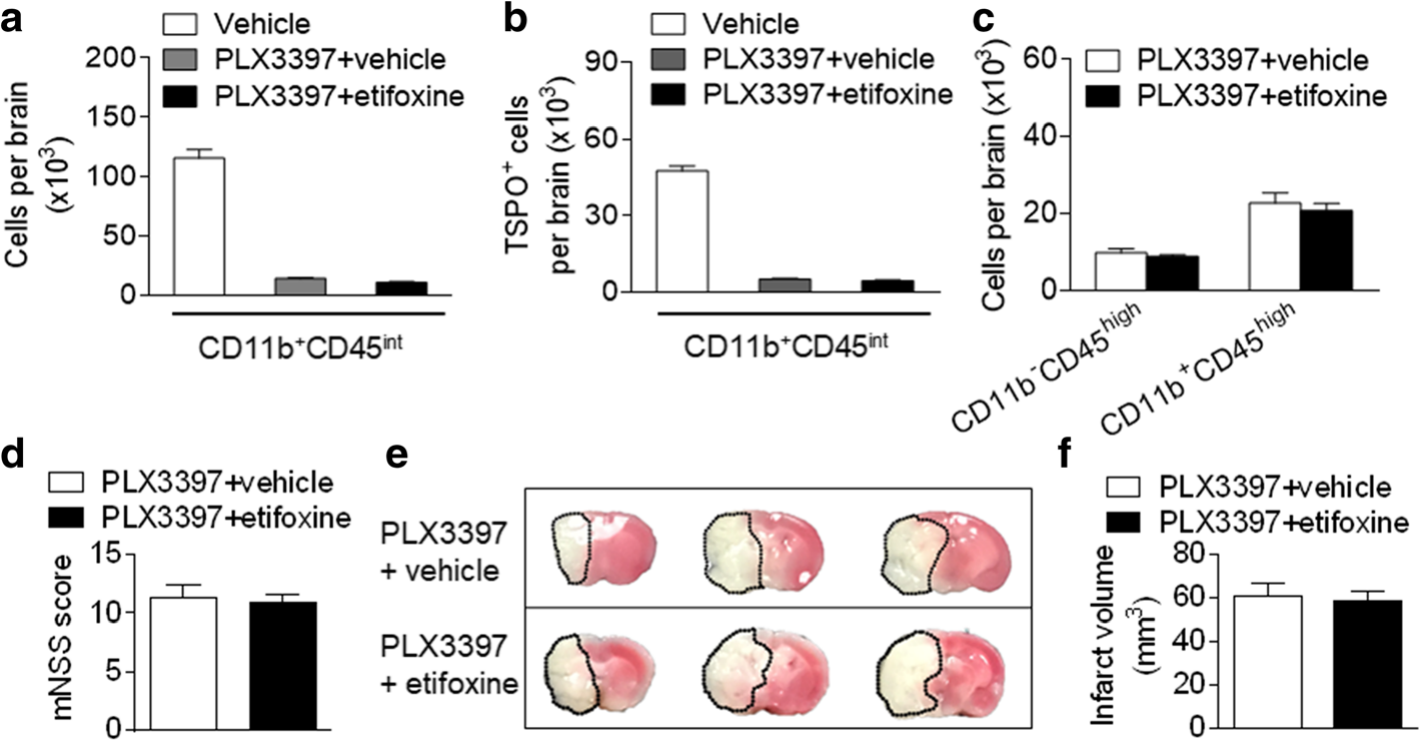
Microglia contribute to the protective effect of etifoxine. C57BL/6 mice received daily treatment of the colony stimulator factor 1 receptor inhibitor, PLX3397, for 21 days prior to 60 min MCAO. After MCAO and immediately at the onset of reperfusion, mice received a single i.p. injection of etifoxine or vehicle control. At 24 h after MCAO and reperfusion, neurodeficits and infarct volume were determined. **a** Bar graph shows numbers of CD11b^+^ CD45^int^ cells in groups of MCAO mice receiving indicated treatments. **b** Bar graph shows numbers of CD11b^+^ CD45^int^TSPO^+^ cells in groups of MCAO mice receiving indicated treatments. **c** Bar graph shows numbers of CD11b^+^CD45^high^ and CD11b^−^CD45^high^ cells in groups of MCAO mice receiving indicated treatments. **d** Bar graph shows neurodeficits based on mNSS in MCAO mice receiving indicated treatments. **e** TTC-stained images show infarct in mice receiving etifoxine or vehicle after MCAO and reperfusion. **f** Bar graph shows infarct volume in MCAO mice receiving etifoxine or vehicle. *n* = 8 per group. Data were presented as mean ± SEM

Since microglia are a predominant cell subset expressing TSPO after brain ischemia, we sought to determine to what extent microglia may contribute to the protective effect of etifoxine. For this purpose, we depleted microglia using a colony stimulator factor 1 receptor inhibitor, PLX3397 [20]. Mice received PLX3397 treatment for 21 days prior to MCAO. Immediately after MCAO and reperfusion, mice were given etifoxine or vehicle. At 24 h after MCAO and reperfusion, we found that PLX3397 treatment eliminated ~85% of the total number of microglia (CD11b^+^CD45^int^ cells) and TSPO^+^ microglia (Fig. 6a, b). Of interest, etifoxine treatment did not alter the numbers of CD11b^−^CD45^high^ and CD11b^+^CD45^high^ cells in MCAO mice receiving PLX3397 (Fig. 6c). Importantly, the neuroprotection of etifoxine and its ability to reduce infarct volume was attenuated in mice receiving PLX3397 (Fig. 6d, e). These results suggest that microglia contribute to the protective effect of etifoxine against ischemia/reperfusion injury.

## Discussion

This study provides definitive evidence that targeting TSPO alleviates ischemic brain injury. Brain ischemia-induced upregulation of TSPO was predominantly observed in microglia. The TSPO agonist etifoxine reduced neurodeficits and infarct volume. In addition to the finding of attenuated brain inflammation and altered microglia response after etifoxine treatment, the benefit of etifoxine was abolished in mice subjected to depletion of microglia. These results together suggest the therapeutic potential of etifoxine to restrict brain inflammation and provide protection against ischemic/reperfusion injury.

Brain ischemia rapidly activates microglia and these cells have multiple capabilities including phagocytosis, production of pro-inflammatory or anti-inflammatory factors and antigen presentation, etc. Although there is still debate on the possible detrimental or protective role of microglia in stroke [26–28], the capability such as the production of pro-inflammatory or anti-inflammatory factors of microglia to influence brain inflammation and neural survival provides evidence that microglia can be potential targets to restrict brain inflammation and tissue injury after ischemia. In this study, we found that expression of TSPO primarily occurred in microglia after brain ischemia and that a TSPO ligand etifoxine can alter microglia response and reduce brain injury. These findings support our hypothesis that targeting TSPO may provide protection against ischemia/reperfusion injury via the modulation of microglia response. In support of this notion, depletion of microglia using the colony stimulator factor 1 receptor inhibitor, PLX3397, abolished the benefit of etifoxine after ischemia/reperfusion, suggesting that the protective effect of etifoxine involves the microglia. It is also noteworthy that CD11b^+^CD45^hi^ leukocytes, which include monocytes and macrophages, also express TSPO after brain ischemia, albeit to a much lesser extent as compared to microglia. Thus, there is a possibility that etifoxine may act on these CD11b^+^CD45^hi^ leukocytes to reduce brain inflammation and neural injury. Therefore, the operating mechanisms by which etifoxine restricts brain inflammation after stroke onset merits future investigations.

Results in this study are consistent with previous studies that show that TSPO ligands can provide protection against neural injury [10, 13–15]. Indeed, we show that etifoxine treatment significantly reduced the production of pro-inflammatory factors after stroke onset. These results are similar with previous reports demonstrating that TSPO ligands provide neuroprotective effects and limit brain inflammation [10, 13–15]. In those studies, reduced expression of the pro-inflammatory cytokines and improved neuronal survival were seen in several models of neural injury [10, 13–15].

Etifoxine has been shown as an enhancer of neurosteroid synthesis [4]. Etifoxine can also facilitate the production of pregnenolone, progesterone, and allopregnanolone that can confer protection against neural injury [29–32]. Reportedly, progesterone treatment following brain ischemia in rodents has provided neuroprotection against ischemic brain injury [33, 34], suggesting the possibility that the protective effect of the TSPO ligand, etifoxine, may involve the local production of neurosteroids that may contribute to limiting deleterious brain inflammation after stroke onset. In addition, neurosteroids like progesterone are also found to be a positive allosteric modulator of gamma aminobutyric acid A (GABAA) receptors [31]. Considering the excessive glutamatergic activities after brain ischemia that result in excitoxicity [35] and the expression of GABAA receptor on microglia/lymphocytes [36–39], the potentiation of GABAergic activities may reduce the pro-inflammatory activities of these immune cells, and thus to attenuate brain inflammation and neuronal death after ischemia. Future investigations are necessary to determine these possibilities.

For clinical translation, while TSPO ligands have been widely used to image neuroinflammation in multiple neuropathological conditions such as Alzheimer’s disease, Parkinson’s disease, and multiple sclerosis, there is no such imaging technique currently available to monitor brain inflammation after stroke. The low expression level of TSPO under physiology condition in the brain regions coupled with the dramatic upregulation induced by brain ischemia enables TSPO to serve as a suitable candidate to monitor inflammation after stroke onset. In addition to the upregulation of TSPO, the protective effect provided by TSPO ligands such as etifoxine against brain infarction suggests a therapeutic potential of targeting TSPO in ischemic stroke.

## Conclusions

We demonstrate that the TSPO ligand etifoxine attenuates neuroinflammation and brain injury after ischemia/reperfusion. Our data suggest that TSPO ligands may serve as a promising candidate for further investigation in ischemic stroke.

## Abbreviations

CBF: Cerebral blood flow
CNS: Central nervous system
DAPI: 4′,6-Diamidino-2-phenylindole
GABAA: Gamma aminobutyric acid A
Iba1: Ionized calcium-binding adaptor molecule 1
IL-6: Interleukin 6
MCAO: Middle cerebral artery occlusion
mNSS: Modified neurological severity score
PCR: Polymerase chain reaction
TNF-α: Tumor necrosis factor alpha
TSPO: 18-kDa translocator protein
TTC: 2,3,5-Triphenyltetrazolium chloride
